# Case Report: Right phrenic nerve palsy following esophageal atresia repair: a report of two cases and literature review of management strategies

**DOI:** 10.3389/fped.2026.1733741

**Published:** 2026-04-21

**Authors:** Wenyue Liu, Bingliang Li, Xiaoxia Wu, Baohong Zhao, Yuanyuan Jin, Liang Zhao, Hongxia Ren

**Affiliations:** 1 Department of Neonatal Surgery, Shanxi Provincial Children’s Hospital, Taiyuan, China; 2 Department of Pediatrics, Shanxi Medical University, Taiyuan, China

**Keywords:** complication, conservative management, diaphragmatic plication, esophageal atresia, phrenic nerve injury

## Abstract

**Background:**

Phrenic nerve palsy (PNP) following esophageal atresia (EA) repair is an extremely rare complication with limited global experience. This report of two cases, integrated with a literature review, aims to synthesize available evidence to propose a structured management strategy.

**Case presentation:**

We describe two neonates who developed right PNP after type III EA repair. Case 1 was diagnosed with right phrenic nerve palsy on postoperative day (POD) 7 via chest x-ray and ultrasound and achieved full recovery after five weeks of non-invasive respiratory support. Case 2 was diagnosed on POD 14; after six weeks of failed conservative management, the infant underwent successful diaphragmatic plication. Both infants had favorable outcomes at follow-up.

**Conclusion:**

Based on the present cases and a review of all documented literature, we recommend a stepwise approach for managing post-EA repair PNP: Preventively, intraoperative phrenic nerve monitoring should be considered during EA/TEF repair to reduce the risk of iatrogenic injury. For diagnosed cases, we suggest early diagnosis using bedside x-ray and ultrasound, followed by an initial trial of 4–6 weeks of non-invasive support, with diaphragmatic plication reserved for weaning failure.

## 1 Introduction

Congenital esophageal atresia (EA) with or without tracheoesophageal fistula (TEF) is a relatively common neonatal malformation, with an incidence of approximately 1.77–4.17 per 10,000 live births (1, 2). Although surgical repair of EA and/or TEF yields high survival rates and good functional outcomes, postoperative complications such as anastomotic leakage, stenosis, or gastroesophageal reflux are well-recognized and relatively common (3–5). In contrast, PNP following EA repair is extremely rare. To date, only five cases of PNP after EA with TEF repair have been reported in the international literature. To consolidate global experience and refine clinical management, this study presents two new cases of right PNP after EA repair and conducts a literature review. Our aim was to synthesize existing evidence, analyze diagnostic and therapeutic approaches,explore preventive measures—particularly the role of intraoperative nerve monitoring,and propose an evidence-based preventive and management strategy for this rare complication.

## 2 Case presentation

### 2.1 Case 1

**Figure 1:**
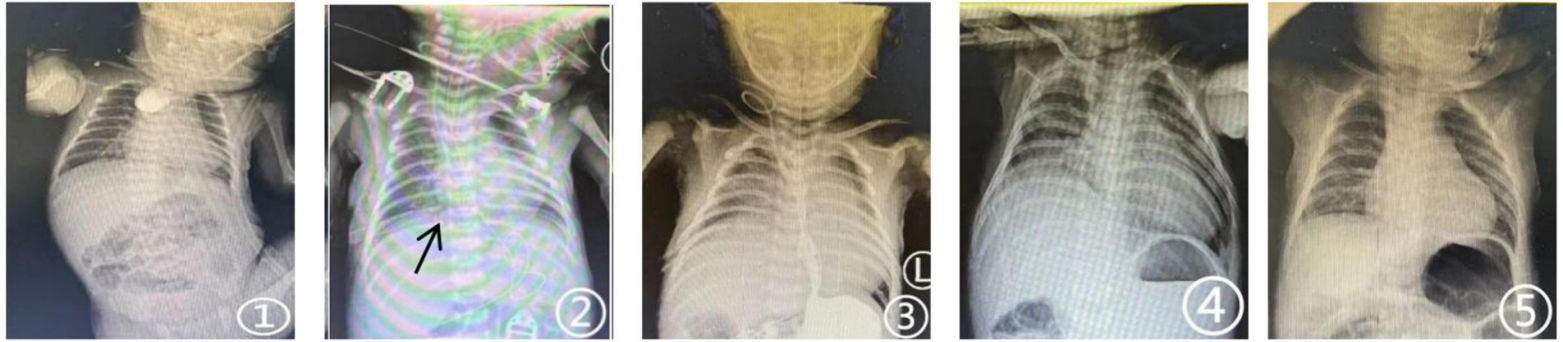
Serial chest x-rays of case 1 (conservative management). **(1)** (Preoperative): Esophagogram showing the proximal esophageal pouch. **(2)** (Postoperative day 3): Bedside chest x-ray showing the chest tube positioned in the right paravertebral mediastinum (arrow) with normal diaphragmatic contour. **(3)** (Postoperative day 7): Bedside chest x-ray revealing significant elevation of the right hemidiaphragm. **(4)** (Postoperative day 42): Chest x-ray showing persistent right hemidiaphragm elevation. **(5)** (1-month follow-up): Chest x-ray demonstrating the right hemidiaphragm largely returned to its anatomical position.

A 1-day-old male neonate (birth weight 3.2 kg, born at 40 weeks via cesarean section) presented with tachypnea and frothy oral secretions. Physical examination revealed respiratory distress with retractions and bilateral crackles. Esophageal atresia was suspected after an 8Fr gastric tube could not be advanced beyond 11 cm. Esophagography confirmed a proximal esophageal pouch at T3 [Figure 1(1)], and chest CT confirmed type III EA with a distal TEF 1.2 cm below the pouch. Echocardiography showed atrial septal defect, patent ductus arteriosus, and pulmonary hypertension.

After three days of preoperative preparation, the infant underwent thoracoscopic EA/TEF repair. The procedure included: right thoracoscopic approach with artificial pneumothorax; azygos vein ligation and TEF suture ligation; mobilization of esophageal segments (1.2 cm gap) and end-to-end anastomosis with 5-0 PDS; mediastinal pleural flap overlay; and chest tube placement. Intraoperative phrenic nerve monitoring was not utilized during this procedure.

Postoperatively, the infant was transferred to NICU. By POD 3, he was weaned to nasal oxygen and started enteral nutrition. Chest x-ray showed pulmonary infiltrates but symmetrical diaphragmatic movement [Figure 1(2)]. On POD 4, he was discharged from NICU, but on POD 7 developed acute respiratory distress with cyanosis. Examination showed diminished right breath sounds and chest movement. Imaging revealed elevated right hemidiaphragm (>3 rib spaces) without anastomotic leakage [Figure 1(3)], and ultrasound confirmed reduced right diaphragmatic mobility (50% of left). Right PNP was diagnosed.

**Figure 2:**
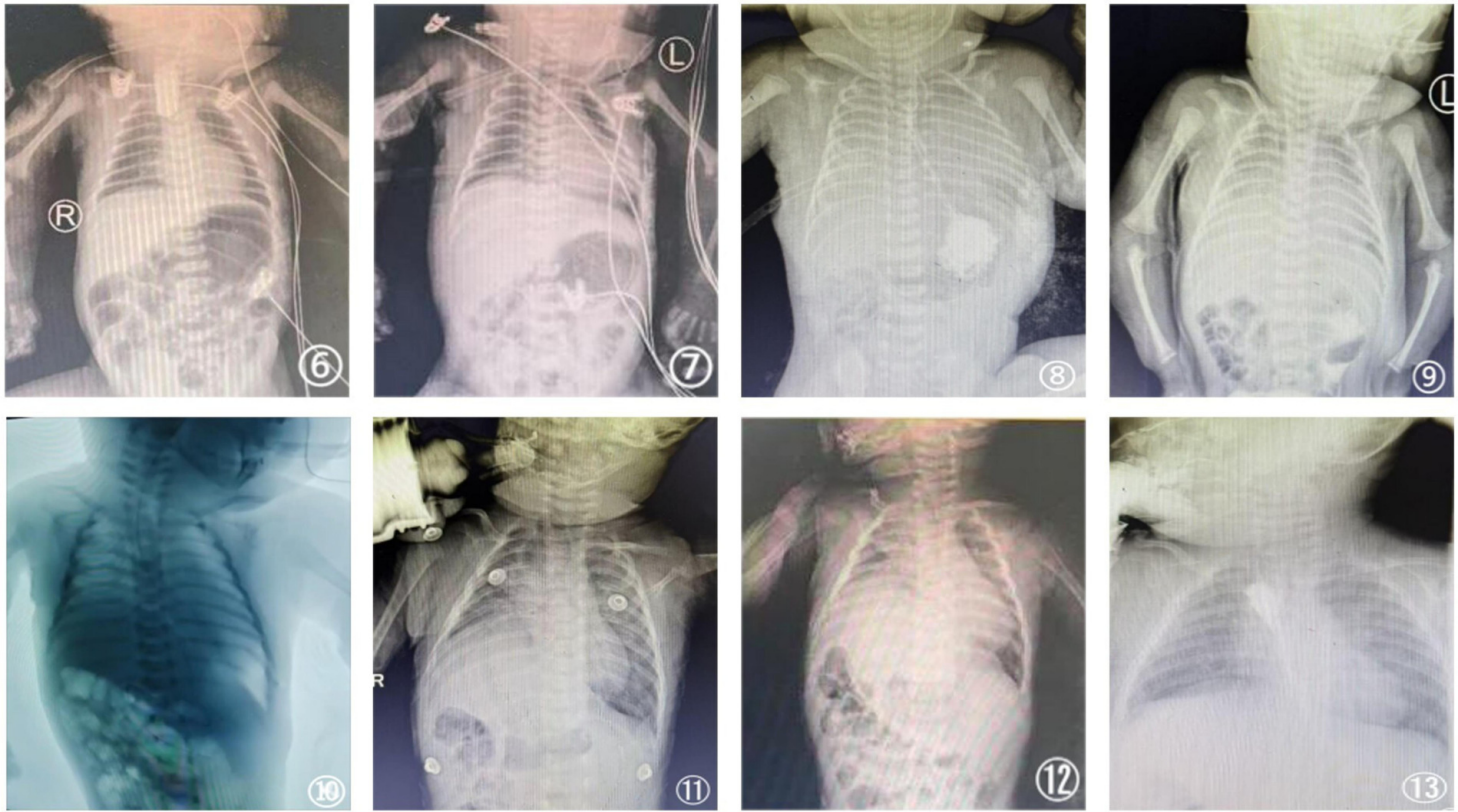
Serial chest imaging studies of case 2 (diaphragmatic plication). **(6)** (Preoperative): Esophagogram showing the proximal esophageal pouch with normal right hemidiaphragm position. **(7)** (Postoperative day 3): Bedside chest x-ray showing normal right hemidiaphragm anatomy. **(8)** (Postoperative day 10): Bedside chest x-ray showing bilateral lung consolidation (“white lung” phenomenon). **(9)** (Postoperative day 14): Bedside chest x-ray revealing new elevation of the right hemidiaphragm. **(10)** Fluoroscopy image demonstrating paradoxical movement of the elevated right hemidiaphragm. **(11)** (1-month post-discharge): Chest x-ray showing persistent diaphragmatic elevation. **(12)** (2-month post-discharge): Chest x-ray confirming ongoing diaphragmatic elevation prior to plication. **(13)** (2-month post-plication): Chest x-ray showing the restored normal anatomical position of the right hemidiaphragm.

The infant did not exhibit paradoxical breathing and never required reintubation or invasive ventilation. Management of concurrent pneumonia and respiratory distress included CPAP, meropenem, and intensive pulmonary care. After 14 days of conservative treatment, he was weaned from CPAP to low-flow nasal oxygen by postoperative day (POD) 21 and discontinued all oxygen support by POD 42, maintaining normal oxygen saturation in room air without respiratory distress. Although chest x-ray at that time still showed right hemidiaphragm elevation [Figure 1(4)], lung infiltrates were resolving, and the infant was discharged on POD 45.

Continued follow-up at 1 month, 3 months, and 6 months post-discharge demonstrated favorable outcomes: growth parameters remained within the normal range for his age; oxygen saturation in room air was maintained at ≥98%. At the 1-month follow-up, chest x-rays showed the right diaphragm had returned to its normal position [Figure 1(5)], and ultrasound indicated gradual recovery of diaphragmatic movement. Pulmonary function tests performed 6 months post-discharge revealed essentially normal results across all parameters. Detailed follow-up data are presented in Table 1.

**Table 1:** Serial follow-up outcomes at 1, 3, and 6 months post-discharge for two infants with phrenic nerve palsy after EA repair.

Parameter	Time point (after discharge)	Case 1 (conservative)	Case 2 (plication)	Normal range
Anthropometrics				
Weight (kg)/Length (cm)	1 month	4.9/54.0	6.9/59.0	-
	3 months	7.0/58.0	7.8/64.0	-
	6 months	8.5/65.0	9.0/68.0	-
Oxygenation				
SpO₂ (Room Air)	All visits	98–100%	97–99%	>95%
Diaphragm Function				
Position (CXR)	1 month	Normal [Figure 1(5)]	Normal [Figure 2(13)]	-
	3/6 months	Normal	Normal	-
Excursion (US)	1 month	80% recovery^a^	90% recovery^a^	R:L ≥ 0.8
	6 months	Full recovery^b^	90% recovery^a^	
Pulmonary Function^c^	6 months			
Tidal volume (mL/kg)		7.2	6.8	6–8
Respiratory rate (/min)		32	35	25–40
Inspiratory time (Ti, sec)		0.52	0.48	0.4–0.6
Expiratory time (Te, sec)		0.68	0.72	0.5–0.8
Ti/Te ratio		0.76	0.67	0.7–1.0
TPTEF (sec)		0.21	0.18	0.15–0.25
TPTEF/Te		0.31	0.26	>0.25
Clinical Status				
Respiratory symptoms	1/3/6 months	None	None	-
Interventions	1 month	Endoscopic dilatation ×1	None	-

SpO₂, peripheral oxygen saturation; CXR, chest x-ray; US, ultrasonography; TPTEF, time to peak tidal expiratory flow (airway resistance index); TPTEF/Te >0.25 (indicates no small airway obstruction).

a Recovery percentage relative to left diaphragm function;.

b Right-to-left excursion ratio=1.0.

c Instrument: Ultrasonic flow sensor (Jaeger MasterScreen BabyBody, Vyaire).

### 2.2 Case 2

A 1-day-old male neonate (gestational age 38 weeks+2 days, birth weight 3.4 kg) presented with cyanosis and vomiting at 17 h after birth. Transfer to our NICU followed an outside hospital diagnosis of type III EA by contrast study, which showed normal bilateral diaphragmatic position [Figure 2(6)]. Upon admission, the intubated infant underwent confirmatory CT showing proximal pouch at T3 with a 1.5 cm gap to the distal fistula and bilateral aspiration pneumonia. Echocardiography revealed patent ductus arteriosus and patent foramen ovale.

On hospital day 3, the infant underwent EA/TEF repair. Initial thoracoscopy was converted to open thoracotomy due to poor surgical exposure. Through a right mini-thoracotomy, the 1.5 cm gap between esophageal ends was bridged with 5-0 PDS anastomosis, and a chest tube was placed.

Postoperative course included mechanical ventilation until successful extubation to nasal oxygen on POD 4. A chest x-ray on POD 3 showed normal right hemidiaphragm position [Figure 2(7)]. On POD 6, acute respiratory distress developed with cyanosis; chest x-ray revealed bilateral “white lung” opacities without anastomotic leakage [Figure 2(8)]. Management included meropenem and CPAP. By POD 10, pulmonary infiltrates improved. On POD 14, chest x-ray showed new right hemidiaphragm elevation [Figure 2(9)]. Bedside ultrasound confirmed poor diaphragmatic movement, and fluoroscopy demonstrated paradoxical inspiration [Figure 2(10)], consistent with right PNP.

The infant exhibited marked paradoxical abdominal breathing and received CPAP for 13 days (until POD 23), followed by nasal oxygen. Serial chest x-rays showed persistent right hemidiaphragm elevation, and the child remained oxygen-dependent due to ventilatory dysfunction. The family opted for discharge against medical advice on POD 32, with continued home oxygen. A one-month post-discharge chest x-ray [Figure 2(11)] showed persistent diaphragmatic elevation, and the parents initially declined surgery (∼6 weeks post-PNP diagnosis). Two months post-discharge, a chest x-ray [Figure 2(12)] continued to show elevation with ongoing oxygen dependence, prompting thoracoscopic right diaphragmatic plication. The child was successfully weaned from oxygen 12 days post-plication and discharged 15 days after surgery.

Continued follow-up at 1 month, 3 months, and 6 months post-discharge demonstrated favorable outcomes: growth parameters remained within the normal range for his age; oxygen saturation in room air was maintained at ≥97%. Imaging findings: At the 1-month follow-up post-plication, chest x-rays [Figure 2(13)] showed the right diaphragm in a normal position, and ultrasound indicated recovery of diaphragmatic movement. Pulmonary function tests performed 6 months post-discharge revealed essentially normal results across all parameters. Detailed follow-up data are presented in Table 1.

## 3 Discussion

The phrenic nerve (C3-C5) innervates the diaphragm and is crucial for respiration (6). Injury can cause hemidiaphragm paralysis. PNP is reported in 1.2–2.4% of thoracic surgeries (7, 8) but is exceptionally rare after EA repair. A literature search up to April 2025 identified only five publications documenting PNP following EA/TEF repair globally (9–13). Among these, four provided complete case data (9–12). Combined with the two cases presented here, a total of six cases with detailed documentation are now available for analysis (Table 2).

**Table 2:** Published cases of phrenic nerve palsy after EA/TEF repair and comparison with the two current cases.

Author (Ref)	Year	BW (g)	Surg. approach	PNP Dx (POD)	Diagnostic method	Treatment & outcome
Haller (9)	1979	NR	Open	NR	CXR	CPAP → Recovered
Man (10)	1982	1,505	Open	10	CXR + Fluoroscopy	IPPV (4W) → CPAP/IMV (6d) → Recovered
Henderson (11)	2010	2,000	Open	5	CXR + Fluoroscopy	CPAP (34d) → Recovered^a^
Pokharkar (12)	2023	NR	Open	3	CXR + US	Oxygen (5w) → Plication → Recovered
Case 1	2025	3,200	Thoracoscopy	7	CXR + US	CPAP (2W) → Nasal O_2_ (3w) → Recovered
Case 2	2025	3,400	Open^b^	14	CXR + US + Fluoroscopy	CPAP (13d) → O_2_ (>8w) → Plication → Recovered

BW, birth weight; Surg, surgical; Dx, diagnosis; POD, postoperative day; CXR, chest x-ray; US, ultrasonography; IPPV, intermittent positive pressure ventilation; IMV, intermittent mandatory ventilation; CPAP, continuous positive airway pressure; w, weeks; d, days; NR, not reported.

a Recovered but died of cardiac disease at 2 months.

b Thoracoscopy converted to open.

Haller et al. (9) first reported one case in 1979. Man et al. (10) described a 1982 case of right diaphragmatic paralysis after thoracotomy for type III EA, with weaning failure from ventilation; recovery occurred after five weeks of respiratory support. Henderson et al. (11) reported in 2010 an infant with difficult weaning, right hemidiaphragm elevation, and paradoxical movement; despite diaphragmatic recovery after six weeks of conservative management, the infant died of congenital heart disease at two months. The most recent report by Pokharkar et al. (12) in 2023 documented right PNP after open repair of long-gap EA/TEF, diagnosed on postoperative day 3 by x-ray and ultrasound; after five weeks of failed conservative weaning, diaphragmatic plication was successful. The addition of our two cases augments this limited global experience.

Analysis of these six cases reveals a clear therapeutic pattern, forming a robust evidence base for management. The majority of patients (4/6, ∼67%) recovered after a period of non-invasive respiratory support. In contrast, a significant minority (2/6, ∼33%), including our Case 2 and the case by Pokharkar et al. (12), required diaphragmatic plication following failed conservative weaning, with successful outcomes. This dichotomy naturally supports a stepwise management strategy.

Regarding diagnosis, methods among the six cases included chest x-ray alone (*n* = 1), x-ray plus fluoroscopy (*n* = 2), and x-ray plus ultrasound (*n* = 3, including our cases).

Generally, mild diaphragmatic paralysis may cause minimal or no symptoms, with many cases in older children or adults being incidental findings. However, neonates are more vulnerable to severe respiratory dysfunction due to weaker intercostal muscles, higher chest wall compliance, greater mediastinal mobility, and greater dependence on diaphragmatic respiration (14, 15). Consistent with this, all well-documented cases—including the six analyzed here—exhibited significant respiratory distress, cyanosis, or paradoxical breathing. These acute postoperative manifestations can mimic common complications such as pneumonia, atelectasis, or anastomotic leakage. Accordingly, Henderson et al. (11) emphasized that PNP should be considered in infants with EA/TEF with difficult ventilator weaning.

Due to the absence of characteristic clinical manifestations, PNP diagnosis relies on imaging. Chest x-ray is the preferred initial test, with high sensitivity (>90%) for detecting diaphragmatic elevation despite limited specificity (15, 16). Fluoroscopy, historically the gold standard, is often impractical in critically ill infants. In contrast, bedside ultrasound emerges as a convenient, radiation-free alternative with comparable diagnostic accuracy (17, 18), and has become a fast and reliable method for evaluating diaphragmatic function in intensive care settings (19, 20). In our cases, recurrent post-extubation respiratory distress initially raised suspicion of pneumonia. However, bedside chest x-ray revealed significant right hemidiaphragm elevation, and subsequent ultrasound confirmed impaired diaphragmatic movement, with paradoxical breathing additionally observed in Case 2. Fluoroscopy later performed in Case 2 provided further confirmation. These experiences highlight that PNP should be included in the differential diagnosis for unexplained weaning failure after EA repair. A combination of bedside chest x-ray and diaphragmatic ultrasound enables rapid diagnosis. While fluoroscopy was utilized in one of our cases and two historical cases, it is not mandatory for confirmation in acute settings, though it may be considered for further validation in stable patients.

The etiology of PNP following EA/TEF repair remains incompletely understood, with intraoperative direct injury being the primary mechanism suggested by previous reports (10–12). This includes nerve avulsion from excessive traction during proximal pouch dissection and thermal injury from electrocautery, risks heightened by the fragility of neonatal mediastinal tissues. In our Case 2, limited visibility after azygos vein ligation may have led to direct phrenic nerve injury from blind cauterization or traction. This scenario highlights the potential value of intraoperative phrenic nerve monitoring, which could provide real-time feedback to the surgeon, alerting them to impending nerve stress and potentially preventing such injury.Furthermore, we propose mediastinal pleural interposition as a potential risk factor, as pleural dissection in Case 1 to prevent fistula recurrence may have injured the phrenic nerve's pericardial branch. Although tissue flap interposition is recommended for recurrent fistula repair (21, 22), its potential for nerve injury during primary surgery remains underrecognized. Meticulous dissection with the aid of intraoperative nerve monitoring could help identify the nerve's course and avoid inadvertent injury during pleural flap creation.Thoracic drain compression constitutes another mechanism; in Case 1, the drain tip was positioned high in the paravertebral mediastinum, consistent with neurocompressive injury as described by Ayalon et al. (23) and supported by subsequent reports (24–26). To prevent these complications, we recommend avoiding extensive electrocautery in poorly visualized areas, ensuring drain placement below the diaphragmatic dome away from nerve pathways, we strongly advocate for the consideration and implementation of intraoperative phrenic nerve monitoring (27) as a proactive measure to reduce the incidence of this debilitating complication.

Based on the collective experience from these six cases, we propose a structured, stepwise management algorithm for PNP following EA repair. Crucially, this algorithm begins with prevention. We advocate for the consideration of intraoperative phrenic nerve monitoring during EA/TEF repair, particularly in complex cases or those with anticipated difficult dissection, to minimize the risk of iatrogenic injury. The subsequent approach is two-phased: First, upon diagnosis, initiate a trial of non-invasive respiratory support for a period of 4–6 weeks. This is supported by the observed recovery in the majority (∼67%) of cases with conservative management, as seen in our Case 1 and historical reports. This period allows for potential spontaneous nerve recovery while minimizing the risks of prolonged invasive ventilation. Second, if the infant remains ventilator- or oxygen-dependent beyond this period, or shows signs of respiratory failure, diaphragmatic plication should be actively considered. This intervention is justified by its documented success in cases where conservative management failed (∼33%), including our Case 2 and the report by Pokharkar et al. (12). Evidence suggests that plication within 30–45 days after birth can significantly reduce ventilator time and hospital stay (28–30), a strategy also supported for phrenic nerve injury following cardiac surgery (8, 31). While early surgery shortens the disease course, the risks of anesthesia and reoperation require careful consideration (32–34). Ultimately, a preventive strategy incorporating intraoperative nerve monitoring holds the greatest promise for avoiding the difficult decision between prolonged conservative management and surgical plication.

This study has several limitations. First, the small sample size (only 6 global cases with full data including ours) limits statistical power and precludes multivariate analysis of risk factors. Second, the lack of intraoperative nerve monitoring data hinders definitive confirmation of the phrenic nerve injury mechanism; future validation through multicenter collaboration is essential. Third, although lung function recovered at 6 months in our cases, long-term follow-up (>2 years) regarding exercise tolerance remains necessary.

## 4 Conclusions

In summary, phrenic nerve palsy, though rare, is a serious complication requiring prompt recognition and structured management following esophageal atresia repair. Prevention is paramount, we suggest that intraoperative phrenic nerve monitoring be considered during EA/TEF repair to mitigate the risk of iatrogenic injury. Based on the synthesis of our two cases and the available global literature, we recommend a standardized approach. Bedside chest x-ray combined with diaphragmatic ultrasound constitutes the preferred diagnostic combination for rapid evaluation. The etiology is multifactorial, involving potential intraoperative injury (from traction, electrocautery, or pleural dissection) and postoperative thoracic drain compression. A stepwise management strategy is advocated: an initial trial of 4–6 weeks of non-invasive respiratory support is warranted, with diaphragmatic plication reserved for cases where weaning fails. This evidence-based algorithm, derived from the aggregation of all reported experiences, provides a practical clinical framework for managing this complex condition.

## Data Availability

The original contributions presented in the study are included in the article/supplementary material, further inquiries can be directed to the corresponding author.
